# Endoscopic removal of a large rectal foreign body using an endoscopic retrograde cholangiopancreatography guidewire snare

**DOI:** 10.1055/a-2589-1152

**Published:** 2025-05-22

**Authors:** Kuangjing Wang, Yuan Wang, Zhengyuan Cheng, Haitao Wang, Min Wang, Yingzhou Shen

**Affiliations:** 1 Department of Gastroenterology, Maanshan People’s Hospital, Maʼanshan, China; 2 74734 Department of Gastroenterology, The First Affiliated Hospital with Nanjing Medical University, Nanjing, China

Rectal foreign bodies are a common presentation in emergency departments, predominantly observed in males and often associated with masturbation or sexual practices 1 2 . Endoscopic removal using a snare is the most common approach, but this method often fails for larger, smoother objects 3 4 . In this report, we report a case of successful removal of a rectal foreign body using a self-made snare, which was created by folding a 0.035-inch, 460-cm guidewire into a loop ( Video 1 ).

Endoscopic removal of a large rectal foreign body using an ERCP guidewire snare.Video 1

A 68-year-old man presented to the emergency department 8 hours after unsuccessful attempts to remove a foreign body he had inserted into his rectum. Abdominal CT and 3D reconstruction revealed a large foreign body lodged in the left colon, approximately 20 cm in length with an enlarged tail segment ( Fig. 1 ). Emergency colonoscopy confirmed its presence 15 cm from the anus.

**Fig. 1:**
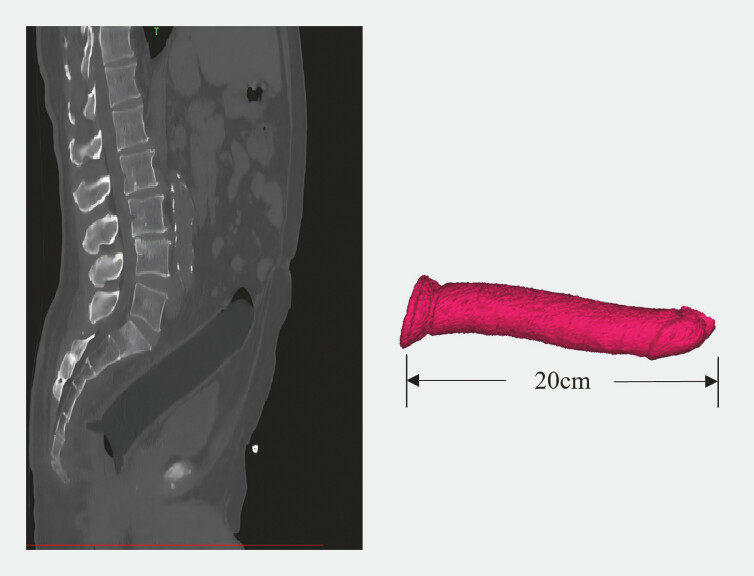
The CT imaging of foreign body. Abdominal CT and 3D reconstruction revealed a large foreign body lodged in the left colon, approximately 20 cm in length with an enlarged tail segment.

Initial attempts to remove the object using foreign body forceps and a snare were unsuccessful due to the object’s smooth surface and the significant resistance it presented. Similarly, a standard 40-mm polypectomy snare failed to grasp the distal end of the object because of its size. To address this, we designed a novel snare device using an endoscopic retrograde cholangiopancreatography (ERCP) guidewire ( Fig. 2 ). A 0.035-inch, 460-cm guidewire was folded, and both ends were inserted retrogradely through the endoscopic accessory channel. This design allowed for adjustable snare diameter based on the foreign body’s size.

**Fig. 2:**
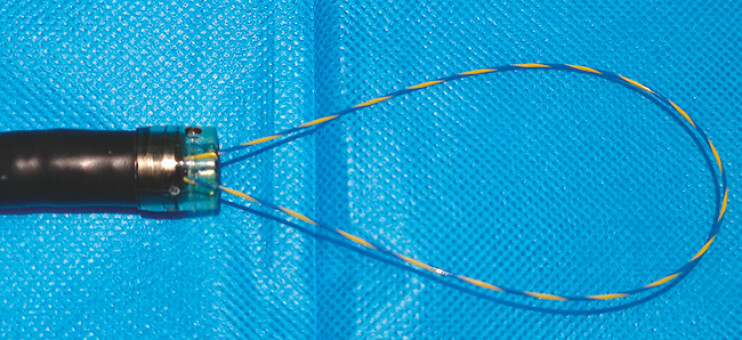
The self-made snare. A 0.035-inch, 460-cm ERCP guidewire was folded, and both ends were inserted retrogradely through the endoscopic accessory channel to form a snare. Abbreviation: ERCP, endoscopic retrograde cholangiopancreatography.

Using this self-designed device, we carefully secured the distal edge of the object and successfully extracted it. The foreign body was identified as a silicone penile prosthesis measuring approximately 200 mm in length and 50 mm in diameter ( Fig. 3 ).

**Fig. 3:**
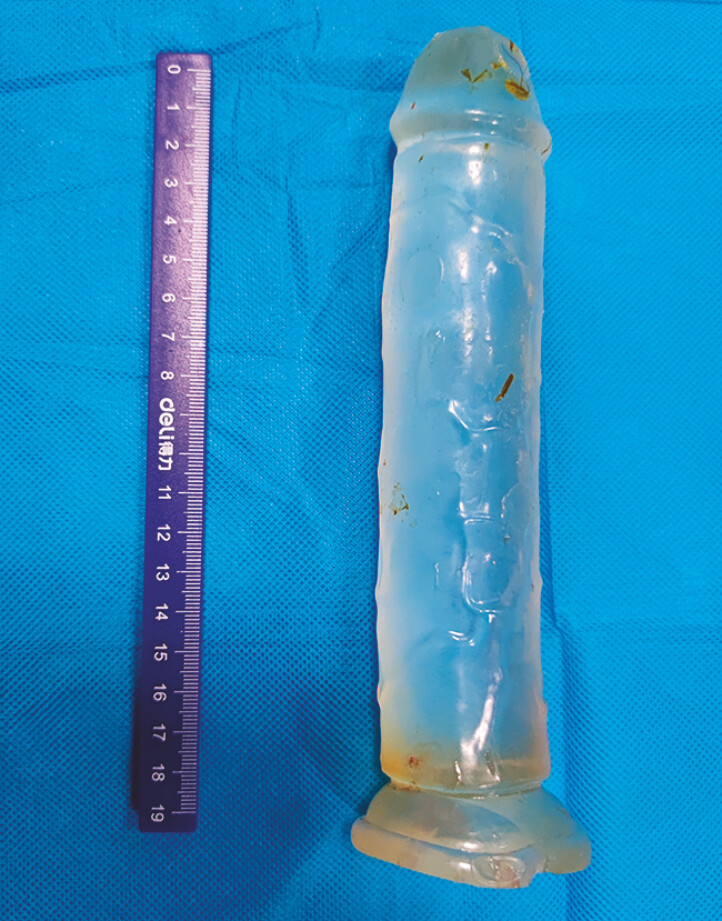
The foreign body. The foreign body was identified as a silicone penile prosthesis measuring approximately 20 cm in length and 5 cm in diameter.

Endoscopy_UCTN_Code_TTT_1AQ_2AH

## Notes

Endoscopy E-Videos https://eref.thieme.de/e-videos
*E-Videos* is an open access online section of the journal *Endoscopy* , reporting on interesting cases and new techniques in gastroenterological endoscopy. All papers include a high-quality video and are published with a Creative Commons CC-BY license. Endoscopy E-Videos qualify for HINARI discounts and waivers and eligibility is automatically checked during the submission process. We grant 100% waivers to articles whose corresponding authors are based in Group A countries and 50% waivers to those who are based in Group B countries as classified by Research4Life (see: https://www.research4life.org/access/eligibility/ ). This section has its own submission website at https://mc.manuscriptcentral.com/e-videos .
